# Antagonistic Effect of a Salivary Proline-Rich Peptide on the Cytosolic Ca^2+^ Mobilization Induced by Progesterone in Oral Squamous Cancer Cells

**DOI:** 10.1371/journal.pone.0147925

**Published:** 2016-01-27

**Authors:** Carlo Alberto Palmerini, Michela Mazzoni, Giorgia Radicioni, Valeria Marzano, Letizia Granieri, Federica Iavarone, Renato Longhi, Irene Messana, Tiziana Cabras, Maria Teresa Sanna, Massimo Castagnola, Alberto Vitali

**Affiliations:** 1 Dipartimento di Scienze Agrarie Alimentari ed Ambientali, Unità di Ricerca di Biochimica e Biologia Molecolare, Perugia, Italy; 2 Istituto di Biochimica e Biochimica Clinica, Facoltà di Medicina, Catholic University, Roma, Italy; 3 Istituto per la Chimica del Riconoscimento Molecolare, Italian National Research Council, Milan, Italy; 4 Dipartimento di Scienze della Vita e dell’Ambiente, University of Cagliari, Cittadella Universitaria, Monserrato, Cagliari, Italy; 5 Istituto per la Chimica del Riconoscimento Molecolare, Italian National Research Council, Rome, UoS Rome, Italy; Lunenfeld-Tanenbaum Research Institute, CANADA

## Abstract

A salivary proline-rich peptide of 1932 Da showed a dose-dependent antagonistic effect on the cytosolic Ca^2+^ mobilization induced by progesterone in a tongue squamous carcinoma cell line. Structure-activity studies showed that the activity of the peptide resides in the C-terminal region characterized by a proline stretch flanked by basic residues. Furthermore, lack of activity of the retro-inverso peptide analogue suggested the involvement of stereospecific recognition. Mass spectrometry-based shotgun analysis, combined with Western blotting tests and biochemical data obtained with the Progesterone Receptor Membrane Component 1 (PGRMC1) inhibitor AG205, showed strong evidence that p1932 performs its modulatory action through an interaction with the progesterone receptor PGRMC1, which is predominantly expressed in this cell line and, clearly, plays a role in progesterone induced Ca^2+^ response. Thus, our results point to p1932 as a modulator of the transduction signal pathway mediated by this protein and, given a well-established involvement of PGRMC1 in tumorigenesis, highlight a possible therapeutic potential of p1932 for the treatment of oral cancer.

## Introduction

Saliva is a mixed fluid, which is secreted by the major and minor salivary glands located in the oral cavity [1], and it exerts various protective and digestive functions [2]. These functions are connected to saliva dynamic composition [3, 4] and for this reason, significant efforts have been devoted to the definition of the salivary proteome [5] with more than 2500 different proteins already been identified [6]. These can be divided into proteins secreted from salivary glands, which include 400–500 components corresponding to approximately 90% of the whole salivary proteome weight, and proteins deriving from other sources (more than 2000) which account for less than 10%.

Proteins and peptides deriving from salivary gland secretion include mucins, α-amylases, histatins, statherin, P-B peptide, S-cystatins, lipases, lipid transporter (lipocalin), and proline-rich proteins (PRPs), divided in acidic (aPRPs), basic (bPRPs) and glycosylated basic (gPRPs) [4]. The attribution of specific functions to the diverse components and families is demanding, probably because each family exerts multiple and integrated functions, and because salivary proteins are randomly dispersed in solution [7–11].

bPRPs, the expression product of four multi-allelic loci named PRB1-4, are a very heterogeneous family of peptides due to extensive polymorphisms, alternative splicing and post-translational modifications. After secretion, bPRPs is partially cleaved into smaller peptides ranging from 8 to 25 amino acid residues by different exogenous proteases [12]. These peptides have peculiar primary sequences which often overlap each other giving rise to a wide set of similar molecules, in some cases only differing by one residue.

One of these peptides (1932 Da, p1932) was patented for its strong anti-viral activity [PCT/IB2012/050415] and was recently found to be internalized within oral mucosal cells [13]. Several experiments performed in order to evaluate biological effects of the peptide evidenced its influence on calcium homeostasis inside cells. The modulation of cytosolic Ca^2+^ concentration ([Ca^2+^]_c_) is an important event occurring in a cell since Ca^2+^ concentration mediates many biological events, such as cell proliferation, differentiation, metabolism, muscle contraction, apoptosis and immune response [14], as well as the exocytosis of synaptic vesicles in the release of neurotransmitters [15].

Calcium homeostasis in mammalian cells is a complex phenomenon and results from the equilibrium between endoplasmic reticulum calcium stores and ion exchanges with the extracellular medium. Progesterone, through genomic and non-genomic effects, plays an important role in the development, growth, and maintenance of female reproductive tissues. In addition, the hormone may impact the physiology of the brain and nervous system, based on its function as a neurosteroid affecting cell survival and growth [16, 17]. The modulation of calcium homeostasis promoted by progesterone is generally regulated through non-genomic mechanisms that involve different types of receptors of progesterone [18, 19]. Interestingly, it should be noted that the hormone is present in saliva in a free form [20].

Here, we describe that p1932 has an antagonistic effect on the cytosolic Ca^2+^ mobilization induced by progesterone in a human squamous cell tongue carcinoma cell line (PE/CA-PJ15). Moreover, we provide strong evidence that the modulatory role of p1932 is enabled via the PGRMC1 receptor, as shown by comparative protein expression studies and biochemical studies performed in the presence of AG-205, a specific inhibitor of PGRMC1 used alone and in combination with p1932. These results, highlight new prospects over the functional implications of salivary proline-rich peptides in cancer cell systems exposed to specific sexual hormones.

## Materials and Methods

### Chemicals

Phosphate Buffer Saline (PBS) and Trypsin-EDTA are products of EuroClone (Padua, Italy). Foetal calf serum (FCS), antibiotics (penicillin and streptomycin), L-glutamine, Hanks Balanced Salt Solution (HBSS), Iscove’s Modified Dulbecco’s Medium (IMDM), Fura 2-AM (Fura 2-acetoxy-methyl-ester), dimethylsulfoxide (DMSO), KCl, MgCl_2_, glucose, Hepes, NaCl, CaCl_2_, EGTA (ethylene glicol-bis (2-amino-ethyl ether)-N,N,N’,N’-tetra-acetic acid), progesterone (P4), Deoxycholic acid, Nonidet P-40 (NP-40), EDTA (2-({2 [Bis (carboxymethyl) amino] ethyl}(carboxymethyl) amino) acetic acid), Tween-20 and AG-205, were purchased from Sigma (Milan, Italy).

### Peptides synthesis

P1932, R.I.-p1932 and fragments F (MER 1–8), C (MER 1-17des Pro), E (MER 1–11), B (MER 1–17) and D (MER 12–20) were assembled on an Applied Biosystem Peptide Synthesizer 433A (Foster City, CA, USA) on a preloaded proline-2-chlorotrityl resin (Novabiochem, Laufelfingen, CH) following the Fmoc-(Nα-9-Fluorenylmethyloxycarbonyl) protocol for stepwise solid phase peptide synthesis [21]. Fmoc-amino acids were from Novabiochem.

All couplings were carried out with 5 fold excess of activated aminoacid in the presence of 10 equivalents of N-ethyldiisopropyl amine, using N-[(dimethylamino)-1-H-1, 2, 3-triazole-[4, 5-β] pyridine-1-ylmethylene]-N-methylmethanaminium hexafluorophosphate N-oxide (HATU, PE Biosystems, Inc., Warrington, UK) as activating agent for the carboxy group. At the end of peptide chain assembly, the peptide was cleaved from the resin by treatment with a mixture of 80% trifluoroacetic acid, 5% water, 5% phenol, 5% thioanisole, 2.5% ethandithiol and 2.5% triisopropylsilane for 3 hours (room temperature), with concomitant side chain deprotection. After filtration of the resin, the peptide was precipitated in cold tert-butylmethyl ether. After centrifugation and washing with tert-butylmethyl ether the peptide was suspended in 5% aqueous acetic acid and lyophilized. Analytical and semipreparative Reversed Phase High Performance Liquid Chromatography (RP-HPLC) was carried out on a Tri Rotar-VI HPLC system equipped with a MD-910 multichannel detector for analytical purposes or with a Uvidec-100-VI variable UV detector for preparative purpose (all from JASCO, Tokyo, Japan). Analytical RP-HPLC was performed on a Jupiter 5 μm C18, 300 Å column (150 x 4.6 mm, Phenomenex, Torrance CA, USA). Semipreparative RP-HPLC was performed a Jupiter 10 μm C18 300 Å (250 x 21.2 mm, Phenomenex, Torrance CA, USA). Linear gradients of acetonitrile in aqueous 0.1% TFA (v/v) were used to elute bound peptide. MALDI-TOF mass spectrometry analysis were performed on a Autoflex workstation (Bruker Daltonics, Bremen, DE) confirming the theoretical mass of the peptide.

### Human PE/CA-PJ15 cell line

PE/CA-PJ15 cells (ECACC, 96121230, Porton Down, UK) [22] were cultivated (37°C, 5% CO_2_) in IMDM medium, containing 2 mM glutamine and penicillin/streptomycin (100 U/mL each), supplemented with 10% inactivated FCS. Cells were subcultured every four day prior to becoming fully confluent.

### Determination of cytosolic concentration of Ca^2+^

FURA-2 AM (2 μL of a 2 mM solution in DMSO) was added to 1 mL PE/CA-PJ15 suspensions (about 10 x 10^6^ cells) obtained after 0.05% trypsin treatment and incubated for 60 min at 37°C, in the dark. Cells were then harvested by centrifugation at 800 g x 10 min and finally suspended in HBSS buffer pH 7.4 (140 mM NaCl, 5.3 mM KCl, 1 mM MgCl_2_, 1 mM CaCl_2_, 5 mM glucose, 25 mM Hepes, adjusted to pH 7.4) to a final cell concentration of 1 x 10^6^ /mL.

An aliquot of this suspension (1 mL) was then centrifuged at 800g x 5 min, after which cells were harvested and suspended in 3 mL of calcium-free HBSS pH 7.4 (140 mM NaCl, 5 mM KCl, 1 mM MgCl_2_, 5 mM glucose, 25 mM HEPES, 0.1 mM EGTA) prior to fluorimetric measurements. Fluorescence was measured with a Perkin-Elmer LS 50 B spectrophotofluorimeter equipped with a double excitation system (ex. 360 and 380 nm, em. 510 nm). Slit widths were set at 1.5 nm for excitation and 3 nm for emission. Cytosolic calcium concentrations ([Ca^2+^]c were calculated as reported [23].

### Proteomic analysis of progestin receptors in PJ15 cells

Protein extracts were obtained by cell lysis with 50 mM Tris-HCl pH 8, 150 mM NaCl, 0.5% Deoxycholic acid, 0.1% SDS, 1% NP-40, 1 mM EDTA pH 8 supplemented with protease inhibitors (RIPA buffer). Proteins were separated on SDS-polyacrylamide gel and, after colloidal Coomassie brilliant blue staining, gel bands of interest were excised, destained with 25 mM NH_4_HCO_3_/ACN 1:1 (v/v), dehydrated with 100% ACN, reduced with 10 mM DTT in 25 mM NH_4_HCO_3_ and alkylated with 55 mM IAA in 25 mM NH_4_HCO_3_. After complete dehydratation, a solution of 0.02 μg/μL trypsin in 25 mM NH_4_HCO_3_ was added, and proteins were digested at 37°C overnight. The reaction was stopped by adding TFA at final concentration of 0.1%. The supernatants containing tryptic peptides were collected along with peptide fragments extracted from the gel with 100% ACN/0.1% TFA in water 3:2 (v/v). Tryptic peptides were analyzed by liquid chromatography-electrospray ionization-tandem mass spectrometry (LC-ESI-MS/MS) on an Ultimate 3000 Micro HPLC apparatus (Dionex, Sunnyvale, CA, USA) equipped with a FLM-3000-Flow manager module directly coupled to a LTQ Orbitrap XL hybrid FT mass spectrometer (Thermo Fisher Scientific, Waltham, MA, USA). Reverse-phase chromatography was performed on a Jupiter C18, 5 μm, 300 Å, 150 ×1.0 mm column (Phenomenex, Torrance, CA, USA) and a 95 min run (gradient 1.6 to 44% acetonitrile in water with 0.1% Formic Acid over 60 min) at a flow rate of 80 μL/min. Mass spectrometric measurements were performed in the positive ion mode with a capillary temperature of 250°C, a sheath gas flow of 40 arbitrary unities, a source voltage of 3.6 kV and a capillary voltage of 48 V. Mass spectra were collected in FT-IT data dependent scan mode (MS scan at 60000 of resolution in the Orbitrap and MS/MS scan on the three most intense peaks in the ion trap). Protein identifications were obtained with the embedded ion accounting algorithm (Sequest HT) of the software Proteome Discoverer (version 1.4, Thermo) and searching a human database (UniProtKB/Swiss-Prot Protein Knowledgebase, release 2013_09 of 18-Sep-13 containing 540958 sequence entries; taxonomical restrictions: Homo sapiens, 20271 sequence entries). The search parameters were 10 ppm tolerance for precursor ions and 0.8 Da for product ions, 1 missed cleavage, carbamydomethylation of cysteine as fixed modification, oxidation of methionine as variable modification and false positive rate under 5% calculated on a decoy database search.

### Progestin receptor western blotting

Protein concentration of cell lysate was determined by the Bio-Rad Protein Assay (Bio-Rad Laboratories, Hercules, CA, USA). Plasma membranes were prepared as described previously [24] with minor modifications. Cells were homogenized in ice-cold homogenization buffer (50 mM Tris-HCl pH 7.4, 0.1 M NaCl, 1 mM EDTA) containing 1% protease inhibitor cocktail (Sigma-Aldrich) by Dounce homogenizer (30 strokes). The homogenate was centrifuged for 10 min at 2000 g and 4°C, followed by centrifugation of the supernatant at 100,000 g for 1 hour at 4°C to pellet plasma membranes, which were resuspended in lysis buffer (50 mM Tris-HCl pH 8.0, 1 mM EDTA, 1% SDS, 1 mM DTT, and protease inhibitor cocktail). The protein quantification was performed using a 2-D Quant Kit protein assay kit (GE Healthcare, Uppsala, Sweden). Proteins were loaded and separated on SDS-polyacrylamide gels, and transferred onto nitrocellulose membranes. Blots were blocked in a blocking solution (PBS, 0.1% Tween-20, 5% (w/v) nonfat dry milk) and incubated overnight at 4°C with the following mouse monoclonal primary antibodies: anti-PGRMC1 (clone C-3, Santa Cruz, CA, USA), anti-nPR (clone 2C11F11, Santa Cruz), anti-mPRα (clone H-76, Santa Cruz) and anti-β-Actin (Clone AC-15, Sigma). Membranes were extensively washed and after incubation with horseradish peroxidase (HRP)-labeled goat anti-mouse or anti-rabbit antibody (Santa Cruz) developed with the ECL plus chemiluminescence detection system (GE Healthcare, UK).

### Statistical analyses

All values are expressed as mean ± SEM (n) and significant levels among groups were assessed by ANOVA and post-hoc Scheffé test. P values below 0.05 were considered statistically significant.

## Results and Discussion

### Antagonistic effect of p1932 peptide on P4 effect

The patented peptide p1932 (NH_2_-^1^GPPPQGGNK^10^PQGPPPPGKPQ—PCT/IB2012/050415) is a fragment deriving from the proteolytic processing of the proline-rich proteins PRB1-L (UniProt/Swiss-Prot, P0428), PRB-2 (P02812), PRB1-M (Q86YA1) and PRB2 (Q7M4Q5) [4]. The sequence of p1932 is repeated four times in PRP1-L and PRB2, and three times in PRB1-M, bringing its average concentration in human saliva around 5–10 μM. Nevertheless, as for many other salivary peptides, the precise role of p1932 in oral physiology and pathology is currently unknown. In a recent paper we have disclosed the ability of p1932 to be internalized within cells of the oral mucosa in a time lapse of few minutes, moreover we demonstrated the lack of cytotoxicity even at concentrations well above those found in saliva for this peptide [13].

In this work we show for the first time the dose-dependent effect of p1932 on the modulation of cytosolic calcium release promoted by progesterone in the PE/CA-PJ15 cell line.

These cells, which may be considered a model of human oral carcinoma cell [25], were purposely chosen to investigate the possible impact of p1932 in the presence of progesterone receptors previously associated with prognostic significance over head-and neck cancer [26].

**Fig 1:**
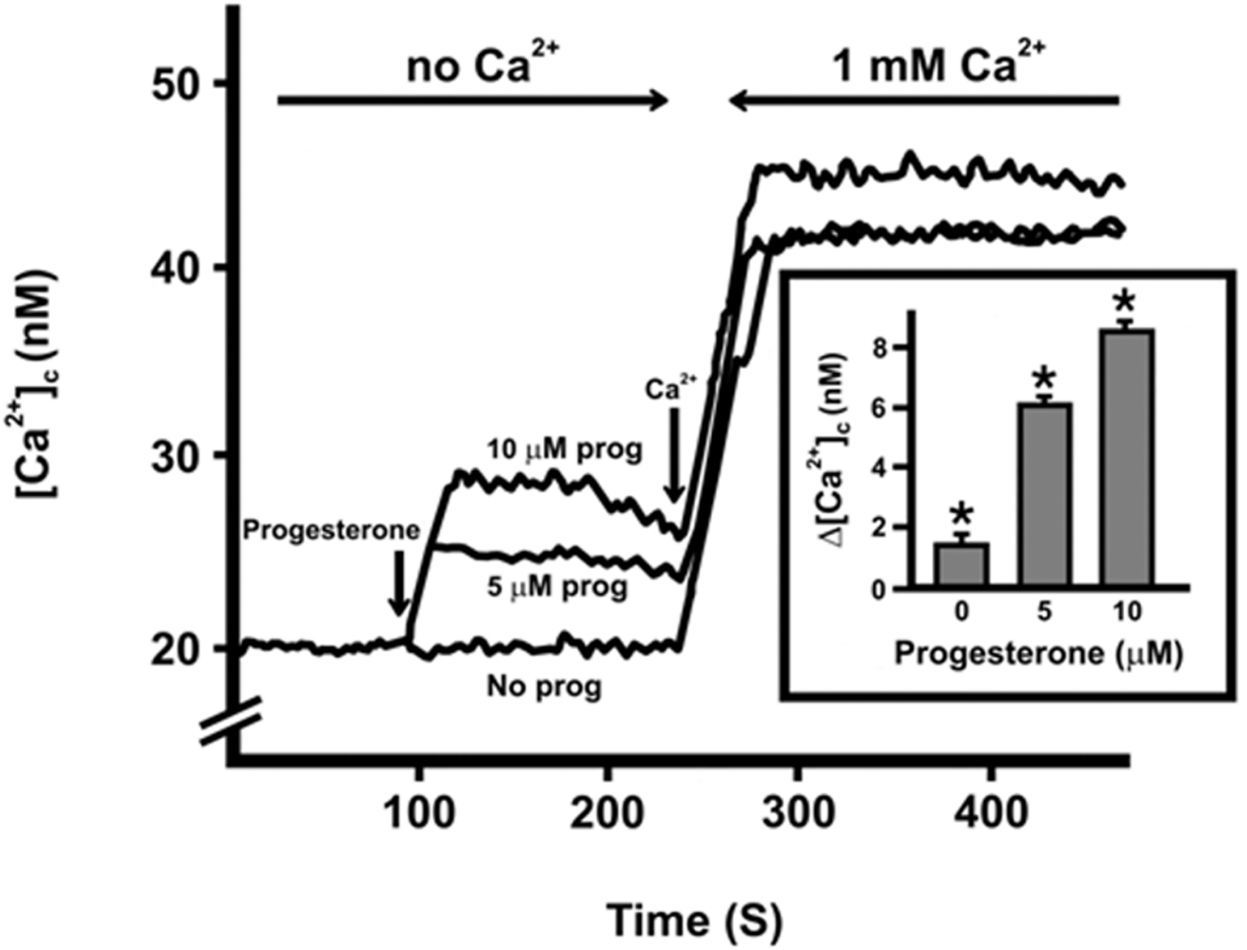
Time-course of [Ca^2+^]c produced by progesterone. The figure illustrates the result of a typical experiment. Cells were suspended in HBSS buffer with no calcium. After 250 seconds calcium was added to reach a concentrations of 1 mM. The experiment was repeated 10 times and a similar behavior was always observed. Inset: Maximal increase of [Ca^2+^]c, reported as Δ[Ca^2+^]c, after the addition of increasing amounts of progesterone. The results shown correspond to the means± SEM of 10 experiments. ANOVA. Effects due to progesterone in the absence of calcium: p<0.0001, and in the presence of 1 mM calcium: not significant (not shown in the inset). Post-hoc: Scheffè’s test at p level 0.05. The asterisks shown statistical significance.

The effect of P4 on the PE/CA-PJ15 cells was studied by employing the hormone under two different concentrations (5 and 10 μM) in order to investigate possible dose-related effects. Progesterone (P4) treatment of cells was carried out in the absence of extracellular calcium which was previously removed with 0.1 mM EGTA 0.1 mM. After few seconds upon P4 treatment, an increase of [Ca^2+^]_c_ could be observed at both concentrations (Fig 1). A such rapid increase suggests a rapid non-genomic effect of P4 [27], that can be mediated by different classes of progesterone receptors, such as the classical nuclear progesterone receptor (nPR) in its monomeric form (nPR-A), the Progesterone Receptor Membrane Components 1 (PGRMC1), and the membrane Progesterone Receptor mPR [28].

The [Ca^2+^]_c_ increase was dependent on progesterone concentration, and in the inset of Fig 1 the effects of P4 on [Ca^2+^]_c_ are reported as Δ[Ca^2+^]_c_ (nM). Statistical analysis confirmed the effect of increasing doses of progesterone on [Ca^2+^]_c_ in calcium-free conditions (p<0.0001). When 1 mM Ca^2+^ was added into the medium, a further increase of [Ca^2+^]_c_ was observed (Fig 1), both in the absence and in the presence of progesterone, towards of 42±3 nM (average of 10 determinations ± SEM). This result indicates that [Ca^2+^]_c_ changes upon P4 stimulus are primarily dependent on the release of Ca^2+^ from intracellular stores, in agreement with previous findings [29]. In this regard, the linear relationship observed between [Ca^2+^]_c_ increase and P4 dose, is likely indicative of a direct impact, thus ruling out unspecific effects of P4 on the cell membranes that, ultimately, would lead to intracellular calcium increases [30]. In the following experiments p1932 peptide was used at a lower concentration (1700 nM) with respect to that found in saliva (5–10 μM), taking in account that in physiological conditions, not all the salivary fraction of p1932 peptide is in contact with mucosal cells at the same time.

**Fig 2:**
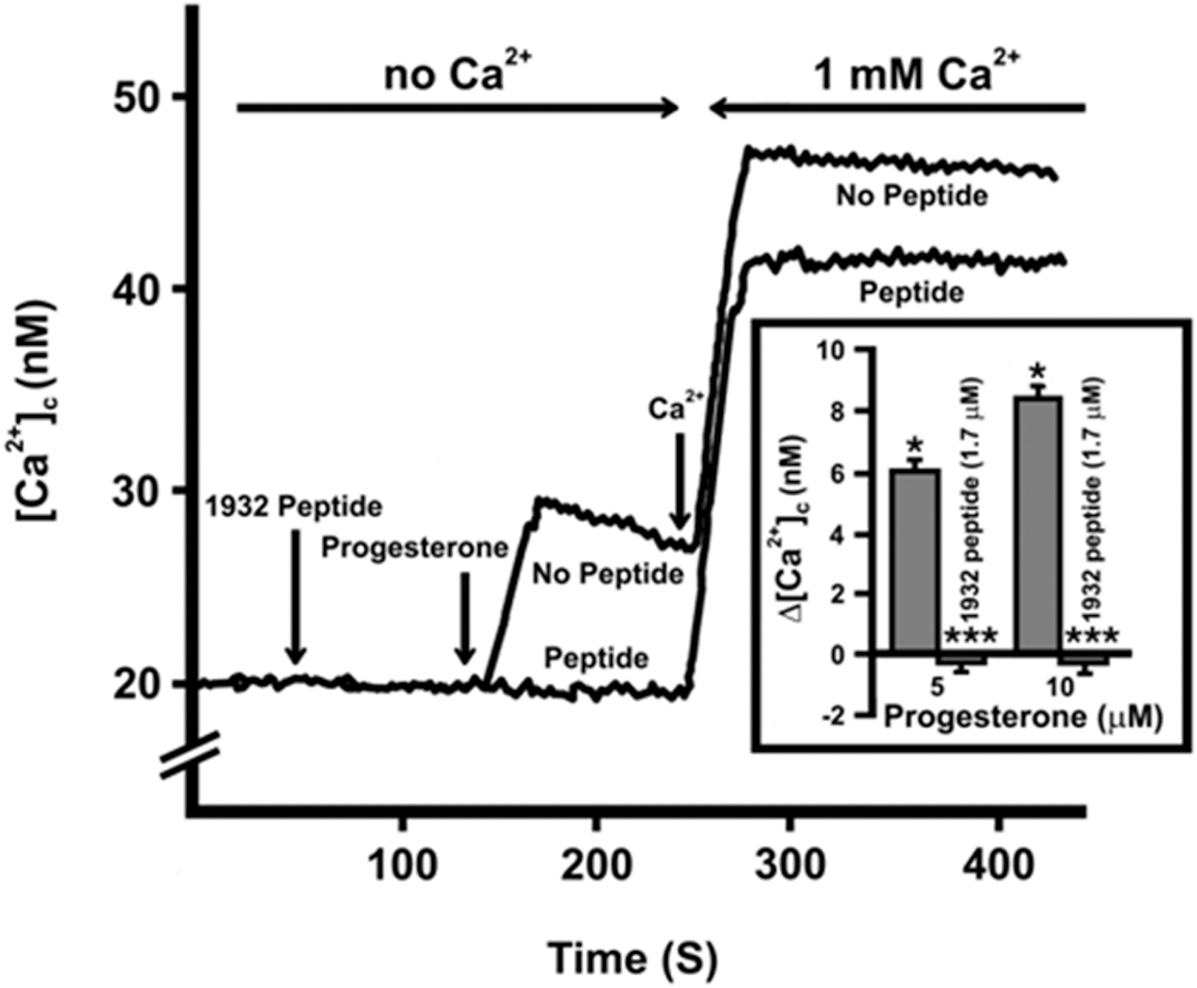
Time-course of [Ca^2+^]_c_ produced by progesterone and/or peptide 1932. The figure illustrates the result of a typical experiment. Cells were suspended in HBSS buffer with no calcium. Peptide (1700 nM) was added two minute before progesterone (10 μM). After 250 s calcium was added to reach a concentrations of 1 mM. Inset: Maximal increase of [Ca^2+^]_c_, reported as Δ[Ca^2+^]_c_, after the addition of increasing amounts of progesterone. The results shown correspond to the means± SEM of 10 experiments ANOVA. Effects due to progesterone in the absence of calcium: p<0.0001 and in the presence of 1 mM calcium: not significant (not shown in the inset). *Post-hoc*: Scheffè’s test at p level 0.05. The asterisks shown statistical significance.

**Fig 3:**
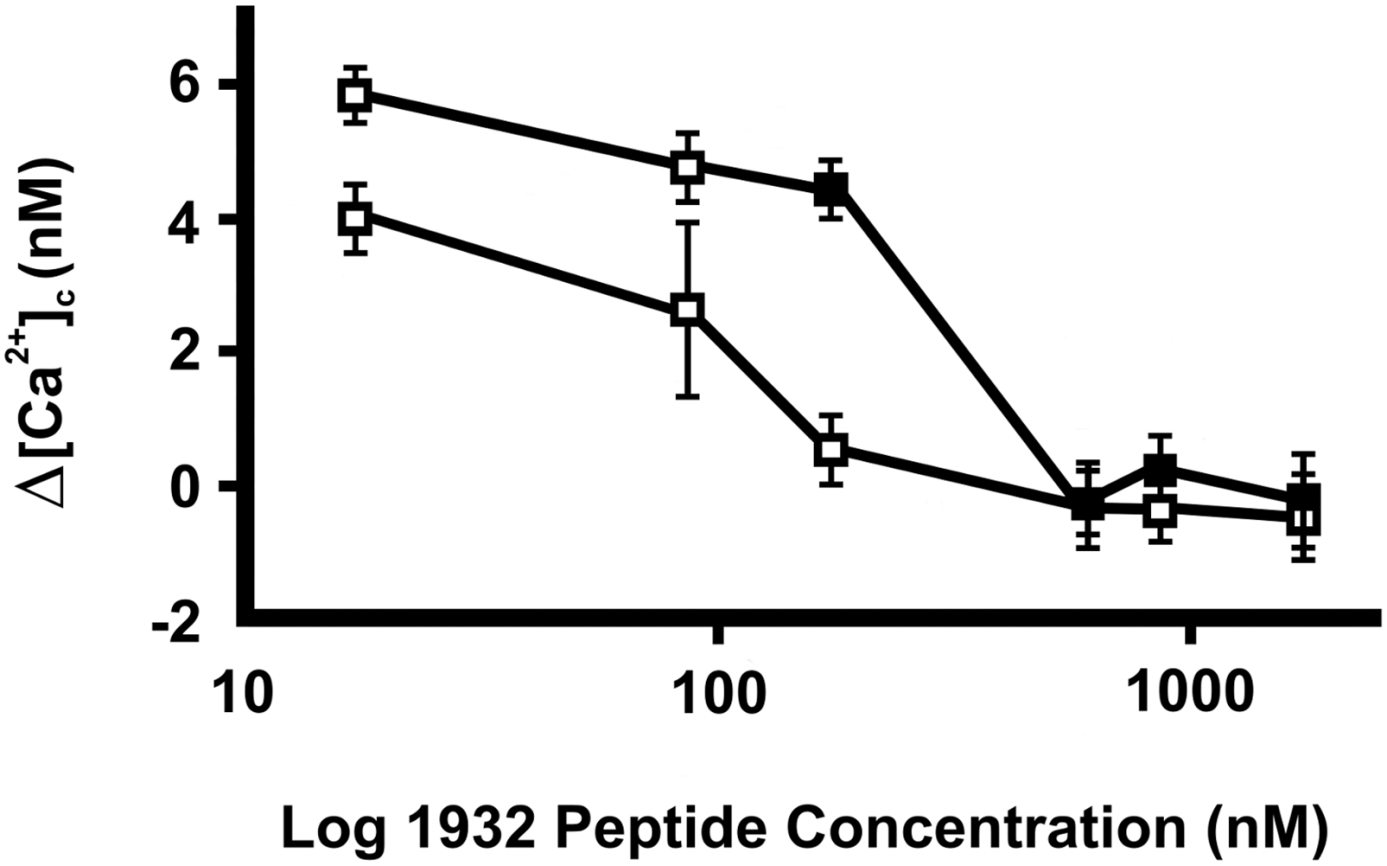
Effects of 1932 salivary peptide concentrations on [Ca^2+^]c increase produced by progesterone. After 120 seconds upon the addition of the peptide (17–1700 nM), progesterone (10 μM [black squares] or 5 μM [white squares]) was also dispensed. Data (reported as Δ[Ca^2+^]c) report the means ± SEM of 10 experiments ANOVA. Effects of progesterone: p<0.001 and of peptide concentration: p<0.001.

Salivary peptide p1932 alone (1700 nM) had no effects on [Ca^2+^]_c_ in PE/CA-PJ15 cells, either in the absence or in the presence of Ca^2+^ in the medium. However, when added 2 minutes before P4 treatment in calcium-free conditions, p1932 completely quenched the effect of progesterone effects (both at 5 and 10 μM concentrations) (Fig 2, inset). In addition, we observed a dose-dependent effect when the p1932 salivary peptide was tested at various concentrations in the 17–1700 nM range (Fig 3). In particular, the upon progesterone-mediated inhibitory effects (5–10 μM) were effectively linear up to the highest concentration (1700 nM) of peptide used.

**Fig 4:**
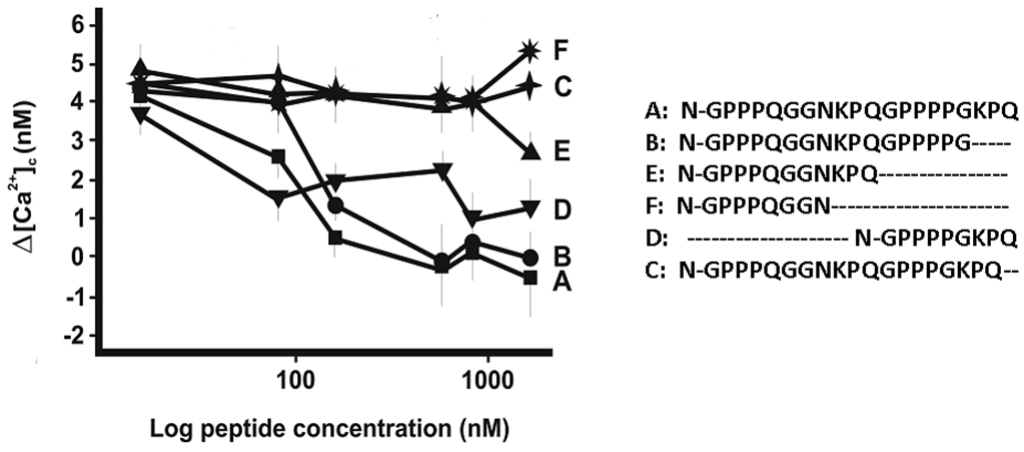
Effects of 1932 salivary peptide and its fragments on [Ca^2+^]c increase produced by progesterone. After the addition of either peptide or its fragments at different concentrations (17–1700 nM), 5 μM progesterone was also added to calcium-free tissue culture medium. Data (reported as Δ[Ca^2+^]c), report the means± SEM of 10 experiments. ANOVA: Effects of peptide: p<0.001 and of concentration: p<0.001. Post-hoc: (scheffée p = 0.05). Homogeneous peptide subsets: 1. A (p1932), B (MER 1–17) and D (MER 12–20); 2. C (MER 1–17 des Pro), E (MER 1–11) and F (MER 1–8).

In order to establish the minimal structural requirements to express the antagonistic effect, we synthesized different fragments of p1932, and performed experiments at 1700 nM to investigate the relationship between fragment sequences and biological activity. Fragments C (MER 1–17 des Pro), E (MER 1–11), and F (MER 1–8) showed a considerable lower antagonizing activity as compared to the intact peptide (Fig 4). Conversely, fragment B (MER 1–17), and fragment D (MER 12–20), resulted as active as p1932 peptide. Interestingly, the MER 1–17 des Pro fragment, which lacks one proline residue, had no inhibitory effect indicating a high degree of specificity with the putative molecular target of p1932 [31]. Finally, the retro-inverso (R.I.) form of p1932 when tested over the 17–1700 nM range of concentrations, showed no antagonistic effect (data not shown), suggesting the involvement of a stereo-specific mechanism of recognition. These results highlight the association of the C-terminal part of the peptide, containing the 12-GPPPPGKPQ-20 proline-rich sequence, with full inhibitory effect on [Ca^2+^]_c_ increase.

### Analysis of Progesterone receptors

**Fig 5:**
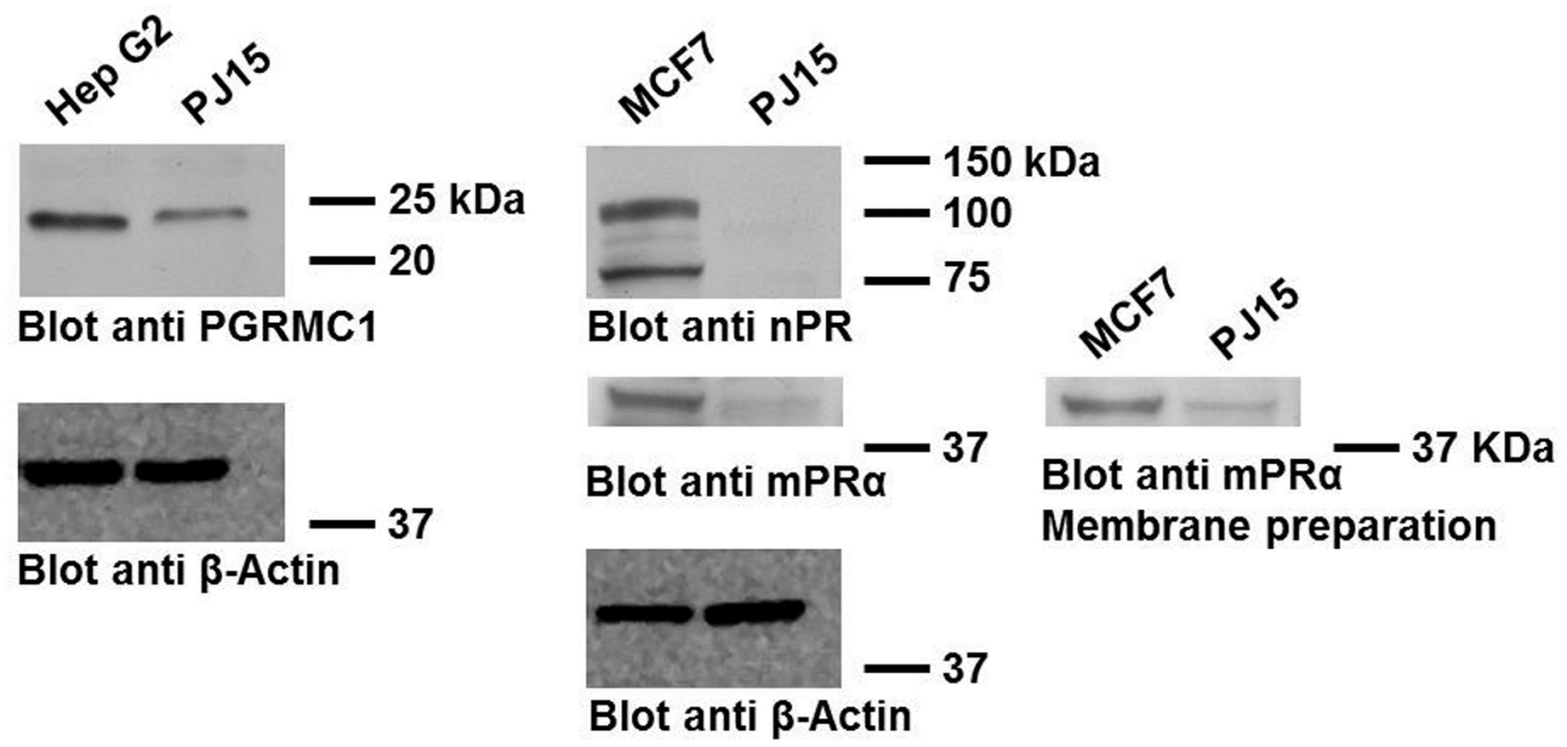
Immunoblotting validation of progesterone receptors expression. PGRMC1, nPR and mPRα proteins levels were revealed with specific antibodies in PE/CA PJ15 cellular extracts obtained lysing cells with RIPA buffer. Blot anti mPRα was performed also on membrane preparation. Proteins extracts from HepG2 and MCF7 cells were analyzed as positive controls (PGRMC1: 21.67 kDa, http://www.uniprot.org/uniprot/O00264; nPR: 82.29 kDa isoform A, 98.98 kDa isoform B, http://www.uniprot.org/uniprot/P06401; mPRα: 39.72 kDa, http://www.uniprot.org/uniprot/Q86WK9). Equal protein loading (50 μg) was confirmed by β-Actin expression.

Proteomic analysis of PJ15 cells was performed in order to demonstrate the presence of the three classes of putative progestin receptors described in the literature: nPR, PGRMC1, and mPRα. After cell lysis, proteins were separated by SDS-PAGE, after which and the proteins with masses in the 21, 40 and 100 kDa ranges were analyzed by LC-ESI-MS/MS. Under these conditions, we identified only PGRMC1 (Table 1) (21 kDa) in duplicate experiments. PGRMC1, mPRα and nPR expression was also assessed by Western blotting analysis, which confirmed that PGRMC1 is the major progesterone receptor expressed in PE/CA PJ15 cells. Western blots also revealed mPRα, although in to a lesser extent compared to PGRMC1 (Fig 5), thus confirming previous co-immunoprecipitation assessments suggesting that, likely, PGRMC1 and mPRα are functionally linked [32]. Conversely, the nPR receptor was not identified, in agreement with the data of Lukits and coll. obtained with the same cell line (Table 1 and Fig 5) [26].

**Table 1:** PGRMC1 identified by LC-ESI-MS/MS. Listed are the following protein parameters: alphanumeric unique protein sequence identifier (Accession), protein identifier characters with a naming convention (Entry name), Gene name and protein name (Description) provided by UniProtKB protein knowledgebase; the calculated molecular weight of the protein in kilo-Dalton units (MW), the theoretically calculated isoelectric point (cal. pI) and the protein number of amino acids (# AAs); protein identification’s SEQUEST HT Score; percentage of protein sequence covered by identified peptides (Cov); number of peptides unique to the protein (# Unique Peptides); number of the identified peptides matching to the protein (# Peptides); total number of identified peptide sequences (peptide spectrum matches) (# PSMs) obtained by two different experiment (Exp 1 and Exp 2). In the table are listed the PGRMC1 peptides following parameters: the identified amino acidic peptide Sequence; Charge state of the precursor ion; the theoretical protonated monoisotopic peptide mass, in daltons (MH+); mass-to-charge ratio (m/z) of the precursor ion, in daltons; the difference between the theoretical mass of the peptide and the experimental mass of the precursor ion in parts per million (ΔM); retention time of the precursor ion, in minutes (R.T.); the SEQUEST HT cross-correlation score for all candidate peptides queried from the database (Xcorr); number of Missed Cleavages.

Accession: O00264 Entry name: PGRC1_HUMAN Gene name: PGRMC1											
**Description**	**MW [kDa]**	**calc. pI**	**Score Exp 1**	**Score Exp 2**	**Cov Exp 1**	**Cov Exp 1**	**# Unique Peptides Exp 1**	**# Unique Peptides Exp 1**	**# PSMs Exp 1**	**# PSMs Exp 2**	**# AAs**
Membrane-associated progesterone receptor component 1	21.66	4.70	15.77	9.54	33.33	23.08	3	2	6	4	195
**Ion Sequence**	**Charge**	**MH**^**+**^ **[Da]**	**m/z [Da] Exp 1**	**m/z [Da] Exp 2**	**ΔM [ppm] Exp 1**	**ΔM [ppm] Exp 2**	**RT [min] Exp 1**	**RT [min] Exp 2**	**Xcorr Exp 1**	**Xcorr Exp 2**	**# Missed Cleavages**
EALKDEYDDLSDLTAAQQETLSDWESQFTFK	3	3623.65	1208.56	1208.56	-0.48	2.25	50.65	49.98	4.52	3.54	1
GDQPAASGDSDDDEPPPLPR	2	2035.88	1018.45	x	1.27	x	24.83	x	2.70	x	0
FYGPEGPYGVFAGR	2	1516.72	758.87	758.87	0.70	1.51	37.07	36.41	2.57	2.42	0

**Fig 6:**
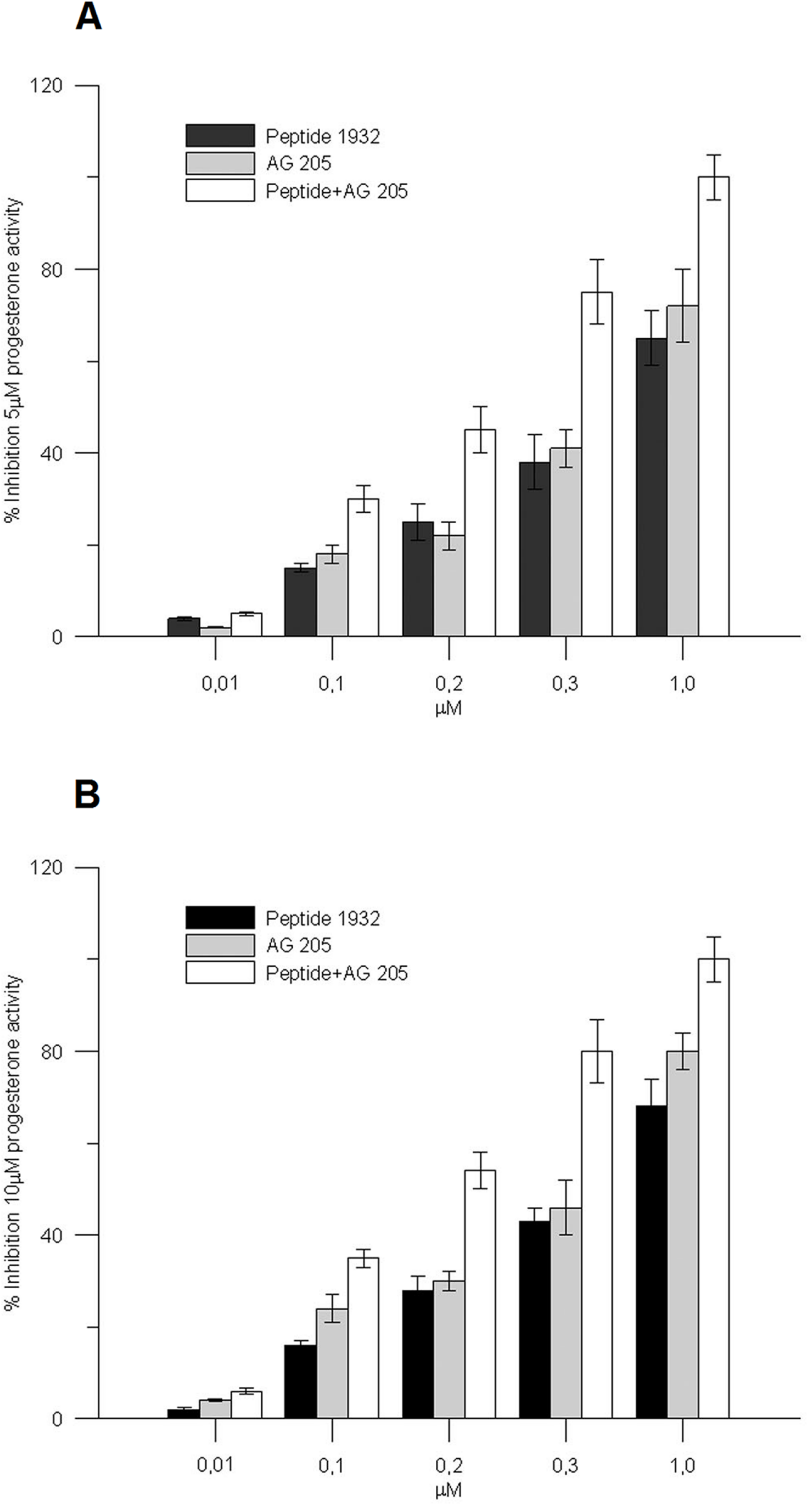
Inhibition experiments with AG205 and p1932 peptide. The experiments were carried out on PE/CA PJ15 labeled with FURA-2 AM. After 50 seconds in the incubation medium, P4 (5 μM, Fig 6A; 10 μM, Fig 6B) was added and the increase in cytosolic calcium as Δ[Ca^2+^]c measured. The PGRMC1 inhibitor AG 205 (0.01–1.0 μM) and/or p1932 peptide (0.01–1.0 μM), was added in the medium 50 seconds prior to 5 or 10 μM progesterone. In the presence of AG205 or p1932 peptide, the effect of P4 (5–10 μM) on Δ[Ca^2+^]c was statistically inhibited in a dose dependent manner. When AG 205 (0.01–1.0 μM) and p1932 peptide (0.01–1.0 μM) were added in combination, the observed inhibitory effect was higher than in samples treated with either agent alone. Each data correspond to the mean± SEM of six independent measurements.

Due to the high expression of PGRMC1 in PE/CA PJ15 cells, we wanted to ascertain its role in the [Ca^2+^]c increase after progesterone stimulus by using the specific inhibitor AG-205 alone and in combination with p1932. AG-205 is known to bind to the heme binding site of this protein thus modifying its spectroscopic properties and functions [33]. The PGRMC1 inhibitor AG 205 (0.01–1.0 μM) or p1932 peptide (0.01–1.0 μM), were introduced in the incubation medium 50 seconds before progesterone (5 or 10 μM). In the presence of AG-205 or p1932 peptide the effect of P4 (5–10 μM) on Δ[Ca^2+^]c was statistically inhibited in a dose dependent manner, thus mimicking the action of p1932 (Fig 6a). Then, AG-205 (0.01–1.0 μM) and p1932 peptide (0.01–1.0 μM) were added simultaneously in the medium 50 seconds before the progesterone. The combination of the two agents at the same doses, led to an inhibitory effect higher on progesterone activity, than that observed for the individual AG-205 or p1932 peptide alone, indicating an additive action of the two molecules (Fig 6b).

The PGRMC1 receptor contains an N-terminal transmembrane domain (72–135 aa) and a cyt-b5 or heme/steroid binding domain [34]; similar features are shared with its homolog PGRMC2. Both proteins are expressed in various human tissues, including heart, liver, and placenta [35], and are overexpressed in many cancer cell types [36–38]. PGRMC1 binds P4 with moderate affinity and mediates P4 anti-mitotic and anti-apoptotic action. It is localized to the plasma membrane, cytoplasm and nuclear membranes, thus explaining its potential involvement in rapid non-genomic progesterone signals. Recently, PGRMC1 was found to be strictly correlated to Sigma-2 receptors [39], despite the existence of some controversial results [40].

The mPRs (MW 40 kDa) were firstly isolated in seatrout [41] and are represented at least by five subtypes i.e. α, β, γ, δ, and ε strictly related to Progestin and adiponectin-Q-receptor (PAQR) family [42]. Their effective presence and nature as progestin receptors have been established after years of debate [43]. These receptors have high affinity for P4 (Kd 5 nM) and may mediate rapid responses as being directly coupled to G proteins [32, 44]. Both PGRMC1 and mPRα receptors are able to rapidly respond to a P4 stimulus, and, consequently determine an increase of [Ca^2+^]_c_ from endoplasmic stores [45, 46]. Considering how these proteins interplay [30], it is possible that they represent the molecular targets of p1932.

**Fig 7:**
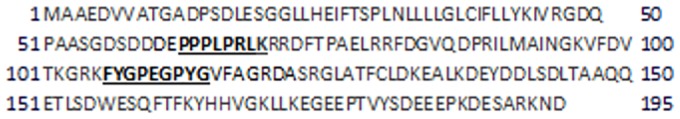
Sequence of human PGRCM1 from Uniprot (O00264). In bold are evidenced the two putative consensus sequences potential target of SH3 domains as obtained by SH3-Hunter web site [50].

The structural features of p1932 strongly suggest this peptide as a potential interactor of the Proline-Rich Recognition Domains (PRDs), and in particular of the SH3 domains [46, 47]. To accomplish the binding between a proline-rich sequence and a SH3 domain, a -PxxP- core must be present in the ligand sequence. Moreover, a basic residue flanking the -PxxP- core is important in defining the interaction specificity determining two types of canonical consensus sequences: the Class I -PxxPxx(R/K) and the Class II -(R/K)xPxxP- [48]. The presence of three -PxxP- consensus cores, in addition to the presence of a Class-II motif in the C-terminal region of p1932 (NH_2_-^1^GPPPQGGNKP^10^QG**P**PP**P**G**KP**Q), in which resides the inhibitory activity, strongly support our hypothesis that p1932 may target SH3 domains. The nPR is known to interact with c-SRC kinase’s SH3 domain through its proline-rich region [49], but the lack of this receptor in PJ15 cells, as resulted from the proteomic and western-blot analyses, rules-out this mechanism. PGRMC1 receptor is instead highly expressed in this cell line, suggesting it is the actual receptor responding to P4 stimuli in PJ15 cells. The transmembrane region of PGRMC1 possesses a putative SH3 target consensus sequences for CrK/Grb2/Abl and Src kinases while the heme binding domain contains consensus sequence for LcK/Abl-P type tyrosine kinases and for ERK1[47], as also predicted by the web software SH3 Hunter [50] (Fig 7).

The role played by PGRMC1 as an intermediate between P4 stimulus and [Ca^2+^] c increase is supported by the effect of its specific inhibitor AG205 [51], in fact, AG205 or peptide p1932 and to a larger extent the combination of the two, greatly lowered the effect of P4. At suboptimal doses, AG205 and p1932 peptide seem to have an additive effect on PGRMC1 inhibition (Fig 6a and 6b), thereby suggesting shared mechanistic properties.

It is therefore possible that, p1932 interferes within the molecular interplay between PGRMC1 and mPRα complex by acting as a competitive inhibitor of the binding between the proline-rich sequences of PGRMC1 and their counterpart, with the latter being, for example, the SH3 domains of specific kinases [52].

Recent studies suggest that PGRMC1/Sigma-2 is a biomarker of cell proliferation and an excellent therapeutic target for inhibiting tumorigenesis [53, 54]. If the role of p1932 as an inhibitor of PGRMC1 signaling pathway can be confirmed in additional cell systems, p1932 would likely emerge as a potential therapeutic agent for the treatment of oral cancer.
